# Mortality difference from *Klebsiella aerogenes* vs *Enterobacter cloacae* bloodstream infections

**DOI:** 10.1099/acmi.0.000421

**Published:** 2023-02-27

**Authors:** Andrew Chou, Richard Sucgang, Richard J. Hamill, Lynn Zechiedrich, Barbara W. Trautner

**Affiliations:** ^1^​ Center for Innovations in Quality, Effectiveness and Safety, Michael E. DeBakey VA Medical Center, 2450 Holcombe Blvd, Suite 01Y, Houston, Texas, USA; ^2^​ Medical Care Line, Infectious Disease Section, Michael E. DeBakey VA Medical Center, Houston, Texas, 2002 Holcombe Blvd 111G/4B370, Houston, Texas, USA; ^3^​ Department of Molecular Virology and Microbiology, Baylor College of Medicine, One Baylor Plaza, Houston, Texas, USA; ^4^​ Margaret M. and Albert B. Alkek Department of Medicine Section of Infectious Disease, Baylor College of Medicine, One Baylor Plaza, Houston, Texas, USA; ^5^​ Center for Health Data Science and Analytics, Houston Methodist Hospital, 6565 Fannin St, Houston, Texas, USA; ^6^​ Verna and Marrs McLean Department of Biochemistry and Molecular Biology, Baylor College of Medicine, One Baylor Plaza, Houston, Texas, USA; ^7^​ Department of Pharmacology and Chemical Biology, Baylor College of Medicine, One Baylor Plaza, Houston, Texas, USA; ^8^​ Section of Health Services Research, Departments of Medicine and Surgery, Baylor College of Medicine, One Baylor Plaza, Houston, Texas, USA

**Keywords:** enterobacterales, genus, clinical, outcome, bacteraemia

## Abstract

Members of the order Enterobacterales, including *Escherichia coli *, *Klebsiella* species and *Enterobacter* species, are important pathogens in healthcare-associated infections. Higher mortality has been reported from infections due to *Klebsiella pneumoniae* than from *E. coli *, but prior studies comparing *Enterobacter aerogenes* (recently renamed *Klebsiella aerogenes *) bacteraemia and *Enterobacter cloacae* complex bacteraemia have yielded conflicting results regarding whether clinical outcomes differ. We found bacteraemia with *K. aerogenes* was independently associated with greater risk of 30-day mortality than bacteraemia with *Enterobacter cloacae* complex.

## Data Summary

The authors confirm all supporting data and protocols have been provided within the article or through supplementary data files. One supplementary figure and two supplementary tables are available with the online version of this article.

## Introduction

*Enterobacter* species, the fourth most common cause of Gram-negative bloodstream infections, are responsible for a potential nationwide ‘second epidemic’ of carbapenem-resistant *Enterobacteriaceae* [1, 2]. A prior study found a higher risk of mortality with *Klebsiella pneumoniae* infections than *Escherichia coli* infections, and concluded that genus and species should be considered as a variable when analysing clinical outcomes in infections due to members of the order Enterobacterales (e.g. *E. coli *, *Klebsiella* spp. and *Enterobacter* spp.) [3]. Analyses comparing *Klebsiella aerogenes* and *Enterobacter cloacae* complex (ECC) have yielded conflicting results (Table S1, available in the online version of this article). Transplant and cancer patients in the Republic of Korea with *K. aerogenes* infections had higher mortality than those with ECC infections [4, 5]. Subsequent studies reported varying results; one study indicated that infections by *K. aerogenes* were associated with poor clinical outcomes relative to ECC, one reported no difference in clinical outcomes between *K. aerogenes* and ECC infections, and one reported higher mortality in patients infected with ECC than with *K. aerogenes* [6–8].

The possibility that clinical outcomes can differ by species is accentuated by the recent nomenclature update that effectively renamed *Enterobacter aerogenes* as *K. aerogenes* [9], but this name change has not been widely accepted by medical microbiologists and it remains unclear whether this nomenclature change should be adopted by clinicians and medical microbiologists [10]. An inducible, chromosomally encoded *ampC* gene is one mechanism of antimicrobial resistance that is shared between the two species. Given the recent attention to *Enterobacter* species and the increasing incidence of carbapenem-resistant *Enterobacter* in healthcare [1], we sought to identify characteristics associated with 30-day all-cause mortality in patients with *Klebsiella */* Enterobacter aerogenes* bacteraemia (KAB) or ECC bacteraemia (ECCB).

## Methods

### Study design

This was a single-centre retrospective cohort study to identify risk factors for 30-day all-cause mortality in patients with *Enterobacter* spp. bloodstream infections. The study included subjects who underwent medical care at the Michael E. DeBakey VA Medical Center in Houston, Texas, USA, from 1 January 2012 to 30 June 2019. This study was approved by the local Institutional Review Board under protocol H-12042.

### Patient population

Study subjects had at least one positive blood culture that grew *K. aerogenes* or ECC and documentation of an infection by the treating clinician. Exclusion criteria included: polymicrobial bloodstream infection, concurrent infection with a different organism, incomplete microbiology testing, post-mortem blood cultures and positive blood cultures deemed to be contaminant by the treating clinician. Only the first eligible episode of KAB or ECCB was included for analysis. Eligible study subjects were identified by querying TheraDoc (Document Storage Systems, Inc., Juno Beach, FL, USA).

### Clinical variables

Extracted electronic health record data included: age, sex, date of death or last follow-up, setting of treatment at onset of bloodstream infection, neutropenia (as defined as an absolute neutrophil count <500 cells µl^−1^), variables included in the Charlson comorbidity index [11–13], variables included in the Pitt bacteraemia score [13, 14], receipt of solid organ or haematopoietic stem cell transplant (defined in accordance with the American Society for Transplantation and Cellular Therapy guidelines) [15] and date of hospital admission. Microbiological data included dates of all bacterial cultures, the specimen type that was cultured (e.g. blood, urine, sputum, etc.), the organism identified in positive cultures and antibiotic susceptibility testing (AST) results. Treatment variables collected included: antibiotic agents administered while hospitalized, outpatient antibiotic prescriptions, source of infection and control of the source of infection (e.g. removal of an infected device, drainage of an abscess, etc.). Short treatment duration was a dichotomized variable defined as antibiotic therapy for ≤10 days [16]. Persistent bacteraemia was defined as a positive blood culture with the same organism >48 h after the index positive blood culture [17].

### Microbiology

Bacterial species were identified using the VITEK MS system [18] and AST was performed using the VITEK 2 system (bioMérieux, Inc., Durham, NC, USA) using cards AST-GN52 and AST-GN67 [19–22]; imipenem and ertapenem were included in both cartridges. Minimum inhibitory concentrations (MICs) were interpreted according to the breakpoints of the Clinical and Laboratory Standards Institute M100-ED29 : 2019 [23]. Discordant carbapenem susceptibility was defined as an isolate that tested as non-susceptible to one carbapenem and susceptible to a different carbapenem [24, 25]. Inappropriate empirical antimicrobial therapy was defined using previously published criteria; specifically, antimicrobial therapy was considered inappropriate if the organism recovered from the blood culture was resistant to the empirically prescribed antimicrobial agent [26].

### Statistical analysis

The primary outcome of the study was 30-day all-cause mortality. Categorical data were compared using the chi-squared test and Fisher’s exact test. Continuous data were compared using the Mann–Whitney U test. Exact tests were used where any estimated occurrence was five or less. The Charlson comorbidity index and Pitt bacteraemia score were analysed as integers, and the unadjusted odds ratio (OR) of each increase of one point was calculated using exact logistic regression. The multivariable logistic regression model included factors that had a *P*<0.20 in univariate analyses and did not have colinearity. Exact multiple logistic regression was used to calculate the adjusted odds ratios (aORs) to identify factors independently associated with mortality. All confidence intervals (CIs) were 95 %, *P* values were two-tailed, and *P*<0.05 was considered statistically significant. Data were analysed using STATA IC version 15.1 (StataCorp, College Station, TX, USA).

## Results

### Study population

Our study found 95 episodes of KAB/ECCB, with 67 episodes meeting study eligibility criteria (Fig. S1). The KAB and ECCB groups were balanced (Table 1) in their median ages, sex, median Charlson comorbidity index scores [11–13], median Pitt bacteraemia scores [13, 14], source control of infection (i.e. was the infectious source appropriately addressed through removal of the infected device or surgical drainage), urinary origin of bacteraemia, infectious diseases service consultation, care in intensive care units, median antibiotic durations, therapy with carbapenems, inappropriate empirical antimicrobial therapy [26] and persistent bacteraemia for >48 h [17]. Blood cultures grew ECC in 79.1 % and *K. aerogenes* in 20.9 % of study subjects. The two groups were similar in the proportion of isolates that were carbapenem intermediate and carbapenem resistant, and those that had discordant carbapenem susceptibilities (see Table S2 for detailed AST results). Sixty-three subjects had follow-up blood cultures. One patient in the *K. aerogenes* group transferred medical facilities during therapy, therefore that subject’s therapy duration was not available for analysis; the patient’s other exposure and outcome variables were accessible and were included in analyses.

**Table 1.** Clinical and microbiological characteristics of patients with *Enterobacter* spp. bacteraemia

Characteristic	Total *n*=67		* E. cloacae * *n*=53		* K. aerogenes * *n*=14		*P* value*
Age, median years (IQR)	68	62–77	67	63–76	68.5	50–84	0.71
Male (%)	65	97	52	98	13	93	0.38
CCI, age adjusted, median (IQR)	7	4–8	6	4–9	7	5–7	0.67
CCI, without age adjustment, median (IQR)	4	2–6	4	2–6	3.5	2–6	0.95
Transplant (%)	3	5	2	4	1	7	0.51
Cancer (%)	23	34	21	40	2	14	0.11
Neutropenia (%)	1	2	1	2	0	0	1.00
PBS, median (IQR)	2	1.5–3.0	2	1–3	3	2–4	0.23
ICU care (%)	22	33	18	34	4	29	1.00
ID consult (%)	25	37	19	36	6	43	0.76
Antibiotic duration, median days (IQR)	13	9–16	13	9–15	14	10–16	0.56
Short treatment duration (%)	23	35	19	36	4	31	1.00
Inappropriate empirical therapy (%)	2	3	2	4	0	0	1.00
Carbapenem therapy (%)	15	22	12	23	3	21	1.00
Urinary source (%)	21	31	16	30	5	36	0.75
Source control (%)	63	94	50	94	13	93	1.00
Persistent bacteraemia (%)	3	5	3	6	0	0	1.00
Discordant carbapenem susceptibility (%)	10	15	6	11	4	29	0.20
30-day mortality (%)	10	15	4	8	6	43	0.004

*Comparing *E. cloacae* vs *K. aerogenes.*

CCI, Charlson comorbidity index; ICU, care in an intensive care unit within 24 hours of first positive blood culture; ID, infectious diseases service; IQR, interquartile range; PBS, Pitt bacteraemia score.

### Associations with 30-day mortality

Thirty-day mortality was greater in patients with KAB than ECCB (42.9 vs 7.6 %). On univariate analysis (Table 2), KAB (OR 9.19, *P*=0.004) and discordant carbapenem susceptibility (OR 5.67, *P*=0.04) were associated with mortality at 30 days, while source control (OR 0.04, *P*=0.009) was protective. Urine as the infection source (OR 0.21, *P*=0.15) and Pitt bacteraemia score (OR 1.31, *P*=0.17) did not meet the cutoff for statistical significance (*P*<0.05) but did meet the threshold for inclusion in the multivariate model (*P*<0.20).

**Table 2.** Variables associated with 30-day mortality

	Odds ratio	95 % CI	*P* value	Adjusted odds ratio	95 % CI	*P* value*
* K. aerogenes *	9.19	1.67–52.65	0.004	10.81	1.24–151.96	0.03
Source control	0.04	<0.01–0.65	0.009	0.03	<0.01–0.77	0.03
Discordant carbapenem susceptibility	5.67	0.88–32.30	0.04	4.31	0.30–112.53	0.45
Urinary source	0.21	<0.01–1.71	0.15	0.07	<0.01–2.27	0.27
PBS	1.31	0.89–1.93	0.17	1.50	0.82–3.07	0.22
CCI, age adjusted	1.16	0.88–1.55	0.32	–	–	–
CCI, without age adjustment	1.14	0.84–1.57	0.43	–	–	–
Cancer	0.79	0.12–3.98	1.00	–	–	–
ICU care	0.86	0.13–4.32	0.57	–	–	–
ID consult	1.14	0.21–5.47	1.00	–	–	–
Short treatment duration	2.11	0.42–10.36	0.30	–	–	–
Inappropriate empirical therapy	<0.01	0–11.70	1.00	–	–	–
Carbapenem therapy	1.61	0.23–8.42	0.68	–	–	–
Persistent bacteraemia	3.79	0.06–79.47	0.34	–	–	–

*Calculated using exact statistical tests.

CCI, Charlson comorbidity index; CI, confidence interval; ICU, care in an intensive care unit within 24 hours of first positive blood culture; ID, infectious diseases service; PBS, Pitt bacteraemia score.

A multivariate logistic regression model using exact methods was generated to identify factors independently associated with 30-day mortality (Table 2). Bacteraemia with *K. aerogenes *, source control, discordant carbapenem susceptibility, urinary source and Pitt bacteraemia score were included in this model. *K. aerogenes* bacteraemia (aOR 10.81, *P*=0.03) and source control (aOR 0.03, *P*=0.03) were the only factors independently associated with 30-day mortality.

Exact statistical methods were used to avoid overfitting with small samples. We also undertook sensitivity analyses to mitigate the risk of model overfitting by removing variables with low event rates or variables that did not meet *P*<0.05 from the models. Infection with KAB remained an independent factor associated with 30-day mortality in these sensitivity analyses, even when source control was removed from the model (aOR 8.58, 95 % CI: 1.28–73.88, *P*=0.02) and in the model using only variables with *P*<0.05 on univariate analysis (aOR 10.73, 95 % CI: 1.46–130.83, *P*=0.02).

## Discussion

In this Veterans Health Administration cohort of patients with KAB/ECB infections, we show that KAB, compared to ECCB, was independently associated with 30-day mortality. Our findings add to the growing body of evidence comparing clinical outcomes between KAB and ECCB (Table S1). Two prior studies found that KAB was more likely (31 vs 16 %; 13 vs 4 %) to be associated with increased mortality than ECCB [4, 5]. A different study [6], however, revealed that KAB was associated with worse clinical outcomes (70 and 40 % for KAB and ECCB, respectively). This latter study used a composite outcome of in-hospital mortality, recurrent bloodstream infection and/or bloodstream infection complication [6]. Two additional studies found either no difference or lower mortality for KAB [7, 8]. One of these publications [7] that found lower mortality associated with KAB than ECCB permitted third-generation cephalosporins as appropriate antimicrobial therapy, a practice not consistent with current guidance from the Infectious Diseases Society of America [27]. The prior studies (Table S1) also examined different clinical endpoints (e.g. 30-day mortality, poor clinical outcome, bloodstream-related mortality, etc.) and controlled for different patient characteristics.

Retrospective studies have inherent limitations. First, our study design is subject to potential unmeasured confounding variables, even though the measured clinical characteristics of the two groups were balanced. By controlling for known clinical and treatment factors (e.g. acute illness severity using Pitt bacteraemia score, chronic comorbidities using the Charlson comorbidity index, antimicrobial class/duration, etc.), we attempted to mitigate the risk of unmeasured confounding variables. Second, we reduced the risk of type I errors that could arise from a small-to-medium size study [28] by using exact statistical methods (i.e*.* Fisher’s exact test, exact logistic regression) rather than using asymptotic measures [29, 30], and by applying nonparametric and semiparametric tests. Third, the study also may have been underpowered to detect other potential variables associated with 30-day mortality, although their effect sizes would be expected to be less than what we detected and to have less clinical and laboratory importance. Larger studies with more diverse patient populations, particularly women and younger patients, are still needed.

In summary, we found that bloodstream infection by *K. aerogenes *, compared to bloodstream infection by ECC, was independently associated with 30-day mortality. Overall, these data are supportive, but not yet conclusive, that clinical outcomes differ between KAB and ECCB, and that, for now, future studies should include the genus/species of pathogens as variables when analysing clinical outcomes.

## Supplementary Data

Supplementary material 1

## References

[1] Wilson BM, El Chakhtoura NG, Patel S, Saade E, Donskey CJ (2017). Carbapenem-resistant *Enterobacter cloacae* in patients from the US Veterans Health Administration, 2006-2015. Emerg Infect Dis.

[2] Al-Hasan MN, Lahr BD, Eckel-Passow JE, Baddour LM (2011). Temporal trends in *Enterobacter* species bloodstream infection: a population-based study from 1998-2007. Clin Microbiol Infect.

[3] Gutiérrez-Gutiérrez B, Pérez-Galera S, Salamanca E, de Cueto M, Calbo E (2016). A multinational, preregistered cohort study of β-Lactam/β-lactamase inhibitor combinations for treatment of bloodstream infections due to extended-spectrum-β-lactamase-producing *Enterobacteriaceae*. Antimicrob Agents Chemother.

[4] Huh K, Kang C-I, Kim J, Cho SY, Ha YE (2014). Risk factors and treatment outcomes of bloodstream infection caused by extended-spectrum cephalosporin-resistant *Enterobacter* species in adults with cancer. Diagn Microbiol Infect Dis.

[5] Song EH, Park K-H, Jang E-Y, Lee EJ, Chong YP (2010). Comparison of the clinical and microbiologic characteristics of patients with *Enterobacter cloacae* and *Enterobacter aerogenes* bacteremia: a prospective observation study. Diagn Microbiol Infect Dis.

[6] Wesevich A, Sutton G, Ruffin F, Park LP, Fouts DE (2020). Newly named *Klebsiella aerogenes* (formerly *Enterobacter aerogenes*) is associated with poor clinical outcomes relative to other *Enterobacter* species in patients with bloodstream infection. J Clin Microbiol.

[7] Jeon M, Huh K, Ko J-H, Cho SY, Huh HJ (2021). Difference in the clinical outcome of bloodstream infections caused by *Klebsiella aerogenes* and *Enterobacter cloacae* complex. Open Forum Infect Dis.

[8] Álvarez-Marín R, Lepe JA, Gasch-Blasi O, Rodríguez-Martínez JM, Calvo-Montes J (2021). Clinical characteristics and outcome of bacteraemia caused by *Enterobacter cloacae* and *Klebsiella aerogenes*: more similarities than differences. J Glob Antimicrob Resist.

[9] Tindall BJ, Sutton G, Garrity GM (2017). *Enterobacter aerogenes* Hormaeche and Edwards 1960 (Approved Lists 1980) and *Klebsiella mobili*s Bascomb et al. 1971 (Approved Lists 1980) share the same nomenclatural type (ATCC 13048) on the Approved Lists and are homotypic synonyms, with consequences for the name *Klebsiella mobilis* Bascomb et al. 1971 (Approved Lists 1980). Int J Syst Evol Microbiol.

[10] Davin-Regli A, Lavigne JP, Pagès JM (2019). *Enterobacter* spp.: update on taxonomy, clinical aspects, and emerging antimicrobial resistance. Clin Microbiol Rev.

[11] Charlson ME, Pompei P, Ales KL, MacKenzie CR (1987). A new method of classifying prognostic comorbidity in longitudinal studies: development and validation. J Chronic Dis.

[12] Quan H, Sundararajan V, Halfon P, Fong A, Burnand B (2005). Coding algorithms for defining comorbidities in ICD-9-CM and ICD-10 administrative data. Med Care.

[13] Wang M, Earley M, Chen L, Hanson BM, Yu Y (2022). Clinical outcomes and bacterial characteristics of carbapenem-resistant *Klebsiella pneumoniae* complex among patients from different global regions (CRACKLE-2): a prospective, multicentre, cohort study. Lancet Infect Dis.

[14] Paterson DL, Ko W-C, Von Gottberg A, Mohapatra S, Casellas JM (2004). International prospective study of *Klebsiella pneumoniae* bacteremia: implications of extended-spectrum beta-lactamase production in nosocomial Infections. Ann Intern Med.

[15] Kanate AS, Majhail NS, Savani BN, Bredeson C, Champlin RE (2020). Indications for hematopoietic cell transplantation and immune effector cell therapy: guidelines from the American Society for transplantation and cellular therapy. Biol Blood Marrow Transplant.

[16] Giannella M, Pascale R, Toschi A, Ferraro G, Graziano E (2018). Treatment duration for *Escherichia coli* bloodstream infection and outcomes: retrospective single-centre study. Clin Microbiol Infect.

[17] Wiggers JB, Xiong W, Daneman N (2016). Sending repeat cultures: is there a role in the management of bacteremic episodes? (SCRIBE study). BMC Infect Dis.

[18] Richter SS, Sercia L, Branda JA, Burnham C-AD, Bythrow M (2013). Identification of *Enterobacteriaceae* by matrix-assisted laser desorption/ionization time-of-flight mass spectrometry using the VITEK MS system. Eur J Clin Microbiol Infect Dis.

[19] bioMérieux, Inc (2012). bioMérieux Connection Durham. https://www.biomerieux-usa.com/news/connection-archive.

[20] bioMérieux, Inc (2011). bioMérieux Connection Durham. https://www.biomerieux-usa.com/news/connection-archive.

[21] Garrec H, Drieux-Rouzet L, Golmard JL, Jarlier V, Robert J (2011). Comparison of nine phenotypic methods for detection of extended-spectrum beta-lactamase production by *Enterobacteriaceae*. J Clin Microbiol.

[22] Mushtaq A, Chasan R, Nowak MD, Rana M, Ilyas S (2022). Correlation between identification of β-lactamase resistance genes and antimicrobial susceptibility profiles in gram-negative bacteria: a laboratory data analysis. Microbiol Spectr.

[23] CLSI

[24] Zaharatos GJ, Dascal A, Miller MA (2001). Discordant carbapenem susceptibility in *Methylobacterium* species and its application as a method for phenotypic identification. J Clin Microbiol.

[25] Kim YJ, Kim SI, Kim YR, Hong KW, Wie SH (2012). Carbapenem-resistant *Acinetobacter baumannii*: diversity of resistant mechanisms and risk factors for infection. Epidemiol Infect.

[26] Kang C-I, Kim S-H, Park WB, Lee K-D, Kim H-B (2005). Bloodstream infections caused by antibiotic-resistant gram-negative bacilli: risk factors for mortality and impact of inappropriate initial antimicrobial therapy on outcome. Antimicrob Agents Chemother.

[27] Tamma PD, Aitken SL, Bonomo RA, Mathers AJ, van Duin D (2022). Infectious diseases Society of America guidance on the treatment of AmpC β-lactamase-producing Enterobacterales, carbapenem-resistant *Acinetobacter baumannii*, and *Stenotrophomonas maltophilia* Infections. Clin Infect Dis.

[28] Octaria R, Rebeiro PF, Kainer MA (2019). Methodologic considerations for small cohort studies. Clin Infect Dis.

[29] Mehta CR, Patel NR (1995). Exact logistic regression: theory and examples. Stat Med.

[30] Young BE, Fong S-W, Chan Y-H, Mak T-M, Ang LW (2020). Effects of a major deletion in the SARS-CoV-2 genome on the severity of infection and the inflammatory response: an observational cohort study. Lancet.

