# Supplementary material for: Mortality difference from Klebsiella aerogenes vs Enterobacter cloacae bloodstream infections
Source: Access Microbiol. 2023 Feb 27;5(2):acmi000421. doi: 10.1099/acmi.0.000421 (PMC10008348; doi:10.1099/acmi.0.000421)
Supplement: Supplementary material 1 [file acmi-5-421-s001.pdf]

Supplemental Materials

Supplemental Figure

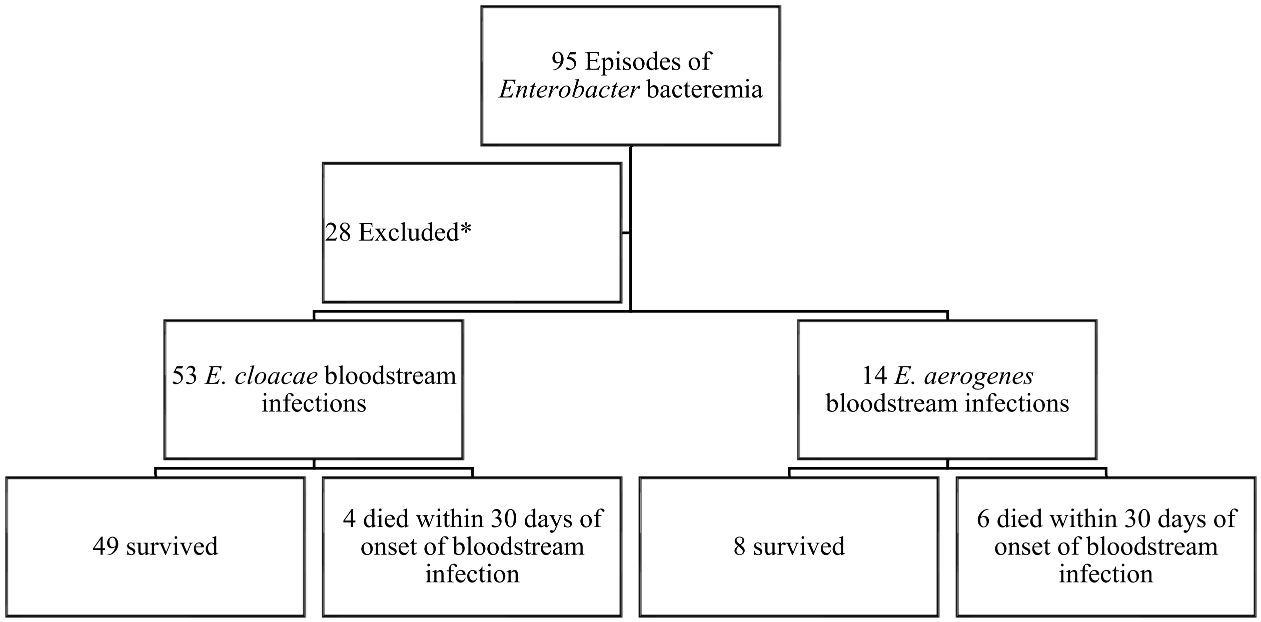

*28 excluded due to: polymicrobial/concurrent infection (20), not first episode during study period (2), VITEK data not available (2), contaminant (2), post-mortem blood culture (2).

Supplemental Table 1: Prior Clinical Studies Comparing *Klebsiella aerogenes* and *Enterobacter cloacae* complex

Variables	Song et al. 2010		Huh et al. 2014	Wesevich et al. 2020		Jeon et al. 2021	Álvarez-Marín et al. 2021
Setting (dates of study)	Single-center<br>Asan Medical Center, Seoul, South Korea (2005–2007)		Single-center<br>Samsung Medical Center, Seoul, South Korea (2004–2011)	Single-center<br>Duke University Medical Center, Durham, United States (2002–2015)		Single-center<br>Samsung Medical Center, Seoul, South Korea (2010–2020)	Multi-center<br>Bellvitge University Hospital (Barcelona), Marqués de Valdecilla University Hospital (Santander), Reina Sofía University Hospital, Virgen del Rocío Hospital (Cordoba), and Virgen Macarena University Hospital (Seville), Spain (2009–2011)
Inclusion criteria	BSI occurred during hospitalization; First eligible BSI per patient		Age ≥ 16 years; Active treatment for cancer or within 1 year of BSI; First eligible BSI per patient	Age ≥ 18 years; First eligible BSI per patient		Age ≥18 years; First eligible BSI per patient	Age >14 years; BSI occurred during hospitalization; First eligible BSI per patient
Exclusion criteria	None		None	Polymicrobial BSI;<br>Blood culture drawn in outpatient setting		Polymicrobial BSI;<br>Blood culture drawn in outpatient setting	None
Number of patients	239		192	150		282	285
Outcome(s)	BSI-related mortality	All-cause 28-day mortality	All-cause 30-day mortality	In-hospital mortality	Poor clinical outcome[a]	All-cause 30-day mortality	All-cause 30-day mortality
% *Enterobacter cloacae* complex	4	8	16	21	40	24	19.4
% *Klebsiella aerogenes*	13	15	31	28	70	11	20.2
Statistical significance of outcome(s)	Adjusted OR 7.74 (*P* = 0.045)	NR	Adjusted OR 2.55 (*P* = 0.04)	Adjusted OR 1.20 (*P* = 0.8)	Adjusted OR 3.30 (*P* = 0.008)	Adjusted OR 2.67 (*P* = 0.003)	Chi-square *P* = 0.87
Patient age, years	ECB: 53 (median)<br>KAB: 57 (median)		57 (mean)	ECB: 57 (median)<br>KAB: 65 (median)		60 (mean)	67 (median)
% Male	56		59	69		61	66
Pitt bacteremia score	NR		1.6	NR		1	1
APACHE II score	NR		NR	7.6		NR	ECB: 19<br>KAB: 14
% Septic shock at BSI presentation	13		NR	NR		NR	9
Charlson score	NR		NR	NR		ECB: 7 (median)<br>KAB: 6 (median)	ECB: 2 (median)<br>KAB: 1 (median)
Malignancy, %	Hematologic: 18		Hematologic: 30	Any malignancy: 40		Any malignancy: 74	Hematologic: 7

	Solid tumor: 44	Solid tumor: 69			Solid tumor: 20
% patients with:					
Diabetes mellitus	13	16	35	24	24
Cardiac disease	NR	5	NR	13	15
Liver disease	32	18	NR	33	47
Renal disease	4	3	19	27	53
Pulmonary disease	NR	3	NR	3	13
Neurological disease	NR	2	NR	13	NR
Transplantation	7	3	11	12	5
Neutropenia or leukopenia	26	38	NR	NR	5
Recent surgery	NR	14	39	19	34
Central venous catheter	NR	41	NR	31	80
Urinary catheter	NR	16	NR	NR	54
Nosocomial acquisition	NR	51	48	77	73
ICU care	21	5	NR	11	35
Urinary source	5	14	15	14	15
Appropriate empiric antibiotics	90	82	NR	78	NR
Resistance to 3rd-generation cephalosporin	51	28	35	29	42
Resistance to 4th-generation cephalosporin	18	6	NR	7	23

[a]As defined by Wesevich et al. (one or more of the following: death prior to discharge, recurrent bloodstream infection, or complication of bloodstream infection)
APACHE II, acute physiology and chronic health evaluation; BSI, bloodstream infection; ECB, *Enterobacter cloacae* complex bacteremia; KAB, *Klebsiella aerogenes* bacteremia; ICU, intensive care unit; NR, not reported; OR, odds ratio

Supplemental Table 2: Carbapenem MICs of *Enterobacter* spp. isolates

	**Ertapenem MIC (µg ml^{-1})**	**Imipenem MIC (µg ml^{-1})**	**Number of isolates**	
**Concordant**	<=0.5	<=0.25	21	57
**Concordant**	<=0.5	<=1	12	
**Concordant**	<=0.5	0.5	13	
**Concordant**	<=0.5	1	11	
**Discordant**	<=0.5	2	6	10
**Discordant**	1	<=1	1	
**Discordant**	2	<=1	1	
**Discordant**	2	0.5	1	
**Discordant**	4	<=0.25	1	

Discordant: defined as discordant carbapenem susceptibilities (see Materials and Methods section)

Supplemental References

Huh K, Kang CI, Kim J, Cho SY, Ha YE, et al. Risk factors and treatment outcomes of bloodstream infection caused by extended-spectrum cephalosporin-resistant Enterobacter species in adults with cancer. *Diagn Microbiol Infect Dis*. 2014; 78:172–7.

Song EH, Park KH, Jang EY, Lee EJ, Chong YP, et al. Comparison of the clinical and microbiologic characteristics of patients with *Enterobacter cloacae* and *Enterobacter aerogenes* bacteremia: a prospective observation study. *Diagn Microbiol Infect Dis*. 2010; 66:436–40.

Wesevich A, Sutton G, Ruffin F, Park LP, Fouts DE, et al. Newly named *Klebsiella aerogenes* (formerly *Enterobacter aerogenes*) is associated with poor clinical outcomes relative to other *Enterobacter* species in patients with bloodstream infection. *J Clin Microbiol*. 2020; 58:e00582–20.

Jeon M, Huh K, Ko JH, Cho SY, Huh HJ, et al. Difference in the clinical outcome of bloodstream infections caused by *Klebsiella aerogenes* and *Enterobacter cloacae* complex. *Open Forum Infect Dis*. 2021; 8:ofab390.

Álvarez-Marín R, Lepe JA, Gasch-Blasi O, Rodríguez-Martínez JM, Calvo-Montes J, et al. Clinical characteristics and outcome of bacteraemia caused by *Enterobacter cloacae* and *Klebsiella aerogenes*: more similarities than differences. *J Glob Antimicrob Resist*. 2021; 25:351–8.
